# Continuous Tracking of Foot Strike Pattern during a Maximal 800-Meter Run

**DOI:** 10.3390/s21175782

**Published:** 2021-08-27

**Authors:** Kathryn A. Farina, Alan R. Needle, Herman van Werkhoven

**Affiliations:** 1 Department of Human Physiology, University of Oregon, Eugene, OR 97403, USA; kfarina@uoregon.edu; 2 Department of Health and Exercise Science, Appalachian State University, Boone, NC 28608, USA; needlear@appstate.edu

**Keywords:** foot strike pattern, foot strike angle, running, track, IMU, curve

## Abstract

(1) Background: Research into foot strike patterns (FSP) has increased due to its potential influence on performance and injury reduction. The purpose of this study was to evaluate changes in FSP throughout a maximal 800-m run using a conformable inertial measurement unit attached to the foot; (2) Methods: Twenty-one subjects (14 female, 7 male; 23.86 ± 4.25 y) completed a maximal 800-m run while foot strike characteristics were continually assessed. Two measures were assessed across 100-m intervals: the percentage of rearfoot strikes (FSP_%RF_), and foot strike angle (FSA). The level of significance was set to *p* ≤ 0.05; (3) Results: There were no differences in FSP_%RF_ throughout the run. Significant differences were seen between curve and straight intervals for FSA_AVE_ (F [1, 20] = 18.663, *p* < 0.001, η_p_^2^ = 0.483); (4) Conclusions: Participants displayed decreased FSA, likely indicating increased plantarflexion, on the curve compared to straight intervals. The analyses of continuous variables, such as FSA, allow for the detection of subtle changes in foot strike characteristics, which is not possible with discrete classifiers, such as FSP_%RF_.

## 1. Introduction

The 800 meter (m) run presents challenges for runners both metabolically and biomechanically due to the unique pacing of the race, in which most elite personal best performances are accomplished by positive pacing or running faster in the first half of the race [1,2,3,4]. There may be biomechanical factors that change throughout the race that may contribute to the runners’ inability to maintain velocity during the second half of the race. Bates and colleagues filmed subjects during a 400 m race and found a significant reduction in velocity throughout caused by a shortening of step length, a reduction in knee drive, and changes in the ability of the limbs to attain the same range of motion by the end of the race [5]. In a 3000 m race, decreases in stride length, increases in ground contact time, and a less perpendicular shank angle upon ground contact (producing a deceleration effect) have been observed [6]. In a study of college-aged physical education students performing an 800 m run, decreases in stride length and frequency, as well as decreases in peak braking and push-off forces, vertical stiffness, and increased center of mass vertical displacement were noted throughout the run [7]. In addition, an observational study of an 800 m race reported increases in ground contact time on the second lap of the race compared to the first, possibly indicative of a shift in foot strike pattern (FSP) [8].

There has recently been an increase in research related to different FSPs during running and how they affect performance and efficiency [9,10,11,12]. FSP is commonly classified as follows: a rear foot (RF) strike, in which the heel of the foot is the first to contact the ground; a mid-foot (MF) strike, in which the middle of the foot hits the ground first; and a forefoot (FF) strike, in which the ball of the foot hits the ground first and the heel rarely, if ever, touches the ground [13]. However, these FSPs exist along a continuum, and it can be difficult to classify all foot strikes into these three rigid categories [13,14]. 

Difficulty in distinguishing between FF and MF strikes has been a primary challenge, leading some investigators to adopt a binary FSP classification of RF and non-RF (NRF) strike patterns, which includes both FF and MF strikes [14,15]. Nonetheless, there are apparent biomechanical differences that occur between FSPs, including ground contact time, ground reaction forces, and lower leg muscle activation [9,16,17,18]. A NRF strike pattern has been proposed to be a preferred choice for faster running and sprinting [19,20]. It has been observed in races from an 800 m run to an ultramarathon that the top finishing runners utilized a NRF strike pattern [8,10,13,21,22].

Running with a NRF strike has been shown to elicit increased plantarflexor muscle activity and may cause higher plantarflexor moments and Achilles tendon strain compared with a RF strike pattern [12,23,24,25]. It is possible the increased demand of plantarflexors of the lower limb with NRF strike running could lead to fatigue of these muscles, which some authors have postulated could be a reason for runners’ inability to maintain a NRF strike pattern for an entire run or race [21,26]. Shifting from a NRF to RF strike pattern has been observed across a range of race distances from 15-min to an ultramarathon [10,21,22,26]. Runners in shorter track races may also be subject to alterations in FSP. Increases in ground contact time have been observed throughout 3000 m, 1500 m, and 800 m races, which could be indicative of shifting from a NRF to RF strike pattern, as a RF strike pattern has been shown to produce increased ground contact times [6,8,9]. However, other investigations have shown no changes in FSP throughout a running event [27,28]. 

Small pace adjustments occur throughout the 800 m run, and consequently could affect FSP at many points during the race [2,29]. Previous investigations have been limited to collecting data at only a few discrete time points during the event using high-speed videography, which would not be able to detect subtle changes in foot strike dynamics throughout the race [7,8,10,13,21,22,28]. More recently, the use of inertial measurement units (IMU) has become increasingly popular to evaluate running kinematics and kinetics outside of the laboratory [30,31,32,33]. IMUs provide the opportunity for a continuous data collection of runners in a real-world environment due to their small size and wireless capabilities, increasing applicability to how runners interact and train in their day-to-day lives. With the use of a small, conformable IMU placed directly on the foot, FSP may be assessed throughout the entirety of a run or race and may allow for a more accurate depiction of alterations in foot dynamics. Therefore, the purpose of this study was to use a conformable IMU attached to the surface of the foot to determine changes in FSP during a maximal 800 m run on a track. We hypothesized there would be a gradual change in foot strike angle causing a potential shift from a NRF to a RF strike throughout the race. 

## 2. Materials and Methods

### 2.1. Participants

Twenty-one runners (Table 1) of varying skill levels (competitive to recreational) were recruited for this study from the surrounding area running community and university. They were healthy men (7) and women (14) (no injuries in the past 3-months, no diabetes, cardiovascular, or renal/kidney disease) and aged 18–35. The participants had to be running at least 10 miles per week and capable of running an 800 m run in under four minutes. Participants completed the 2015 American College of Sports Medicine Exercise Pre-Participation Health Screening Form [34] and provided informed consent, approved by the Appalachian State University Institutional Review Board (IRB).

### 2.2. Experimental Setup

The participants completed the running protocol on a standard 400 m Eurotan outdoor track. The 800 m was performed according to competitive 800 m standards, beginning at the start of the curve in order to simulate a typical race start and finish position.

### 2.3. Instrumentation

**Figure 1:**
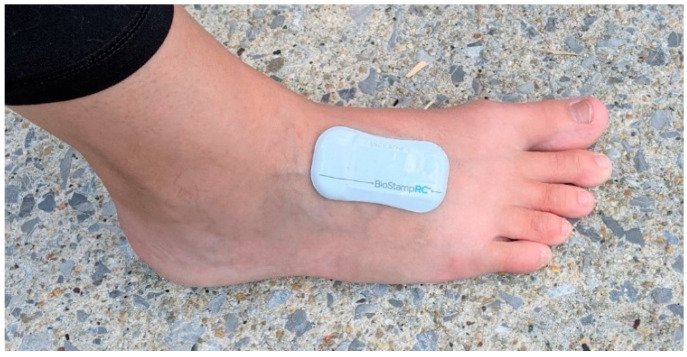
The soft, conformable BiostampRC sensor attached directly to the dorsal surface of the foot before the sock and shoes were put on.

Foot strike characteristics were assessed by means of BiostampRC sensors (mc10, Lexington, MA, USA). These sensors are lightweight, soft and flexible and were placed on the dorsal surface of the foot underneath the shoe and sock of the right foot (Figure 1). 

The sensor contains a 3-dimensional accelerometer (±16 G) and gyroscope (±2000 °/s). Data were collected in on-board memory at an average 250 Hz sampling frequency and later downloaded, after which both accelerometer and gyroscope data were resampled/interpolated at a fixed sampling rate of 1000 Hz. Along with accelerometer and gyroscope data from the one sensor, this study design also attempted to collect muscle activation (EMG) data from the right calf muscles (medial and lateral gastrocnemius) using two separate sensors. For this reason, the accelerometer and gyroscope data were resampled (to 1000 Hz)—to be comparable with the EMG data. Unfortunately, the EMG data proved to be unreliable, and we did not include the muscle activity data here. The sensor used in this study has previously been validated in measuring FSP and determined to have good accuracy for measures of gait parameters and has been validated against traditional goniometer measurements for knee joint angle and range of motion [15,35,36]. To evaluate FSP, accelerometer and gyroscope data from the foot mounted sensor were used to determine two measures of foot strike (see Section 2.5). The sensor was attached with a double-sided sticker pressed firmly onto the skin. Split times were gathered using a stopwatch and taken manually every 100 m of the run. 

### 2.4. Experimental Protocol

Participants were advised of the protocol, their expected effort, and potential risks before selecting a time and date for testing. Upon arrival at the track, each participant was given an informed consent form and an exercise risk assessment questionnaire to complete. Additionally, participants completed a training information form. Age, height, weight, and weekly mileage information were collected. 

The participant was given time to warm-up on the outdoor track or surrounding area. The warm-up was left up to each participant’s discretion based on how they normally warm-up but they were asked to warm-up for a minimum of five minutes, but not more than 15 min. Following the warm-up, the participant was instrumented with the BiostampRC sensor. The participant was then instructed to run the 800 m as if it were a race. Participants were given their split time every 200 m and verbal encouragement was provided. 

### 2.5. Data Processing

**Figure 2:**
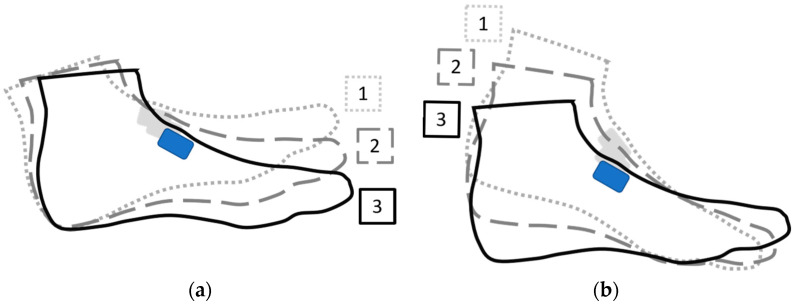
Examples of different foot strike pattern (FSP) and foot strike angle (FSA) classifications. Events: 1—Initial Foot contact; 2—Foot rotation 15 ms after 1; 3—Foot stationary on ground. Clockwise rotation defined as positive angle change, positive angular velocity. (**a**) LEFT HAND SIDE: FSP classification: rearfoot strike (RF)—average angular velocity between 1 and 2 is positive; FSA angle: positive—change in foot (sensor) angle between 1 and 3 is positive; (**b**) RIGHT HAND SIDE: FSP classification: nonrearfoot strike (NRF)—average angular velocity between 1 and 2 is negative.; FSA angle: negative—change in foot (sensor) angle between 1 and 3 is negative.

All data were downloaded and processed via custom MATLAB code (Mathworks, Inc., Natick, MA, USA). Two measures of foot strike were used:

1. Foot Strike Angle (FSA): Calculated as the difference in foot angle at foot contact and the angle when the foot was stationary on the ground, with a larger (more positive) value indicating a more RF strike pattern (Figure 2). The foot angle was measured using integrated angular velocity data (from gyroscope) from the axis aligned with foot sagittal plane. Previous results showed a strong correlation between this measure and 2D videography measure of foot strike angles (r = 0.868) [15]. Average (FSA_AVE_) and standard deviation (FSA_SD_) measures for FSA were calculated for each 100 m interval as well as an average over the entire race. Step-by-step data processing information is provided as Supplementary Materials (see Supplementary Materials—Document S1).
2. Foot Strike Pattern (FSP): A binary classifier indicating rearfoot strike (RF) or non-rearfoot strike (NRF). This binary classifier used an average of 15 ms angular velocity data for the foot, starting at foot contact, to determine type of foot strike. A positive value indicated the foot rotating in one direction, and a negative value indicated the foot rotating in the opposite direction. For our purpose, positive FSP indicated a RF strike and negative indicated a NRF strike (Figure 2). Previous work indicated that this method could accurately distinguish RF from NRF with a 92.2% success rate [15]. FSP measures were used to calculate the percentage of foot strikes that were RF (FSP_%RF_) for each 100 m interval as well as over the entire race. Step-by-step data processing information is provided as Supplementary Materials (see Supplementary Materials—Document S1).

### 2.6. Statistical Analysis

Statistical analyses were performed using SPSS version 26.0 software (IBM Corp., Chicago, IL, USA). Time was analyzed using a two-way repeated measure analysis of variance (ANOVA) to examine two within-subject factors—curve (two levels: straight v. curve interval) and distance (four levels across each 100 m interval of the 800 m run. Similarly, FSA_AVE_ and FSA_SD_ were analyzed using a two-way repeated measure ANOVA to examine two within-subject factors—curve (two levels: straight v. curve interval) and distance (four levels) across each 100 m interval of the 800 m run. In the case data violated the assumptions of sphericity, a Greenhouse–Geisser correction was utilized. Effect sizes were interpreted with partial eta squared (η_p_^2^) where 0.02, 0.08, and 0.14 were considered small, medium, and large effects, respectively. In the case of significant main and interaction effects, Fisher’s LSD comparisons were used post-hoc to determine significant changes. Since percentage of each 100 m interval that utilized a RF strike (FSP_%RF_) was a value presented as a ratio, it was analyzed with a non-parametric Friedman’s test of differences. Further, an a priori level of significance was set at 0.05. 95% confidence interval (CI) for difference is reported for statistically significant post-hoc results.

## 3. Results

### 3.1. Performance

**Figure 3:**
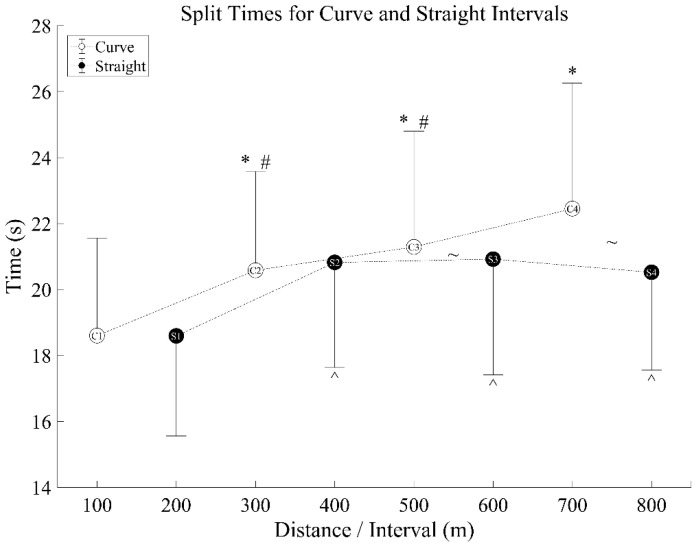
Split times for curve and straight intervals—mean (circles) ± standard deviation bar (vertical bars-shown in one direction only). C1 signifies ‘Curve 1’, S1 signifies ‘Straight 1.’ *—significantly different from C1; #—significantly different from C4; ^—significantly different from S1; ~—significant difference between C3 and S3, and C4 and S4.

The mean final time of all subjects was 163.76 ± 24.11 s. A significant interaction effect was seen for distance and curve (F [1.743, 34.864] = 15.188, *p* < 0.001, η_p_^2^ = 0.432). Post-hoc analysis showed the first curve (first 100 m of the 800 m) was significantly faster than the subsequent three curves (*p* < 0.001, 95% CI [−2.71, −1.25], [−3.74, −1.64], [−5.11, −2.58]), the second and third curves were significantly faster than the fourth curve (2: *p* < 0.001, 95% CI [−2.63, −1.11], 3: *p* = 0.010, 95% CI [−2.00, −0.31]). The first straight (second 100 m of the 800 m) was significantly faster than the second, third, and fourth straights (all *p* < 0.001, 95% CI [−3.04, −1.44], [−3.21, −1.46], [−2.90, −0.98]). When comparing straights and curves at each interval (first curve to first straight, second curve to second straight, etc.), the third straight was faster than the third curve (*p* = 0.039, 95% CI [−0.72, −0.20]), and the fourth straight was faster than the fourth curve (*p* < 0.001, 95% CI [−2.82, −1.03]) (Figure 3). 

### 3.2. Foot Strike Angle (FSA)

**Figure 4:**
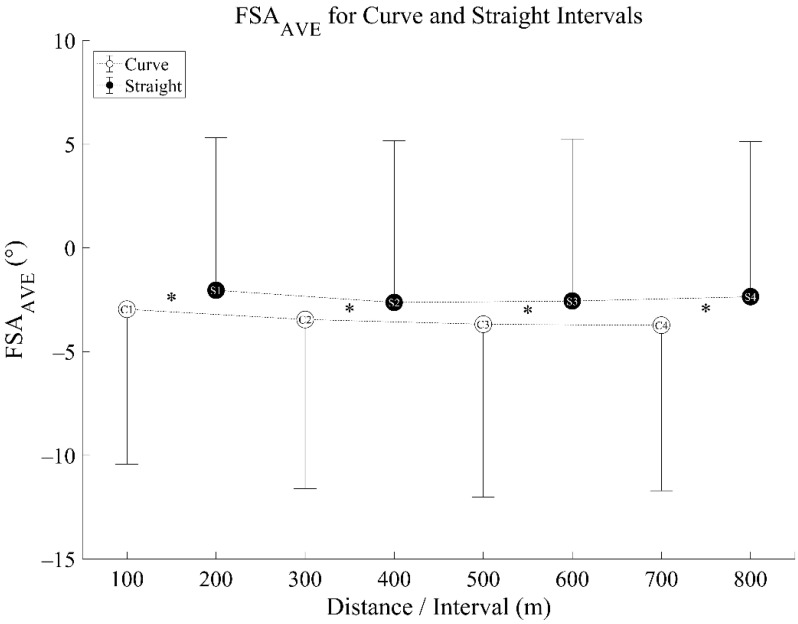
Foot Strike Angle Average (FSA_AVE_) for curve and straight intervals—mean (circles) ± standard deviation bar (vertical lines—shown in one direction only). C1 signifies ‘Curve 1’, S1 signifies ‘Straight 1’. *—significant difference between curves and straights.

FSA_AVE_ revealed no significant distance* curve interaction effect (F [2.029, 40.578] = 1.688, *p* = 0.197, η_p_^2^ = 0.078). There was no significant main effect of distance (F [1.503, 30.053] = 2.125, *p* = 0.147, η_p_^2^ = 0.096), but a significant main effect of curve was observed (F [1, 20] = 18.663, *p* < 0.001, η_p_^2^ = 0.483) with significant differences between curves and straights (*p* < 0.001, 95% CI [−1.57, −0.55]) (Figure 4). Pairwise comparisons revealed smaller (less positive) FSA_AVE_ values during the curves compared to the straights, indicating a more NRF strike angle on the curves.

FSA_SD_ revealed a significant distance*curve interaction effect (F [2.034, 40.677] = 21.964, *p* < 0.001, η_p_^2^ = 0.523). Fisher’s LSD pairwise comparison revealed that the initial curve was significantly more variable than all subsequent curves (all *p* < 0.001; 2: d = 1.04; 3: d = 1.27; 4: d = 1.29), while no differences were observed on straights.

### 3.3. Foot Strike Pattern Classification (FSP)

**Figure 5:**
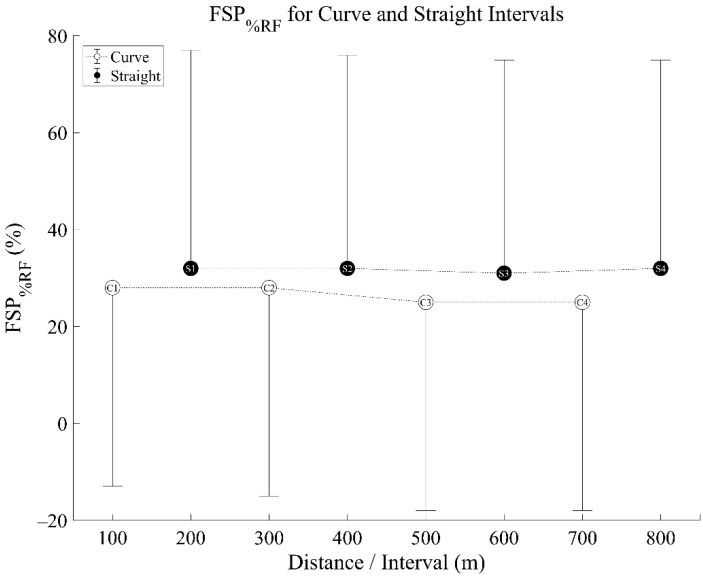
Percentage Rearfoot Strike Pattern (FSP_%RF_) for curve and straight intervals—mean (circles) ± standard deviation bar (vertical lines—shown in one direction only). C1 signifies ‘Curve 1’, S1 signifies ‘Straight 1’. There were no significant differences between distances/intervals.

Comparing the percentage of RF strikes (FSP_%RF_) throughout each segment of the run using a non-parametric Friedman’s analysis of variance revealed no significant differences across eight 100 m segments (χ^2^ =11.01, *p* = 0.138) (Figure 5). There were large individual differences between different subjects, with some subjects not changing foot strike through the race (100% RF or 100% NRF) and others varying foot strike pattern during the race (Supplementary Materials—Table S1).

## 4. Discussion

The main findings of this study revealed that foot strike characteristics did not change significantly and consistently throughout an 800 m run as hypothesized. There was no gradual change in FSA over the course of the race, nor was there a consistent change in FSP over the course of the race. However, there were significant differences seen in FSA between the curves and straights of the track, where a decreased FSA_AVE_ was evident on the curve intervals compared to the straights. 

### 4.1. Performance

A significant decrease in velocity was observed throughout the 800 m run from interval one to eight, however, participants were able to run the last straight interval of the race faster than the previous 100 m. This most likely occurred because the participants had a “kick” during the last 100 m of the run. When looking at the effect of distance and curve on split time, a significant interaction effect was observed. The first curve was run significantly faster than the subsequent three curves. Additionally, the first straight interval was run significantly faster than the following three straight intervals. Similarly, previous observations of 800 m performances have shown the highest velocities during an 800 m run occurred during the first 200 m of the run [2,3,29]. However, contrary to our participants’ results, a decrease in time during the last 100 m of an 800 m run has not been observed, and there is typically a slowing of velocity during the last 100 m [29]. This difference between the present study and previous results for 800 m pacing strategy may be attributed to differences of running in a real race and simulated race, as well as differences in participant caliber. Our participants were a heterogeneous group, consisting of competitive as well as recreational runners of both sexes.

### 4.2. Foot Strike Angle

When looking at FSA, we saw significant differences for the effect of curve, where significantly decreased FSA was evident on the curve compared to the straight intervals. The use of a continuous measure such as FSA, instead of classifiers such as FSP, allows one to see more subtle changes in foot strike characteristics. This is the first study to show differences in FSA between curve and straight running in an 800 m run using an IMU worn throughout the entirety of the run. Previous investigations have also shown differences in foot kinematics when comparing running on a straight and curved surface, where subjects completed repeat trials of running on a curve or straight [37,38,39]. When running on a curve, the outside, right foot has been found to be in a supinated position of at least five degrees more than when running on a straight [38]. Our result of a decrease in FSA on the curve compared to the straight intervals appears to agree with this previous finding [38], as a decreased FSA has been found to be associated with a more plantarflexed foot position, a characteristic present in foot supination [40,41]. Supination is also characterized by frontal and transverse plane foot movements [40], which have also been shown to be affected by curve running [39]. These modifications in foot kinematics appear to be due to the need for the runner to adjust the application of ground reaction force and lean into the curve while running in order to counteract a torque attempting to rotate the runner away from the center of the curve as result of centripetal force generation [38]. The outside foot has been thought to primarily contribute to this generation of centripetal force needed to maintain curved running [39] and appears to accomplish this task through placing the foot in a more supinated position compared to the straight interval [38].

The variation in FSA, measured as standard deviation (FSA_SD_) showed that only the first 100 m, or first curve, was significantly different compared to all other intervals of the run. When running the first curve, participants showed larger variation of FSA. This is not unexpected, since during the first 100 m interval, the runners are changing speed as they accelerate from the stationary start. This changing speed, which is associated with variation in speed, would similarly affect FSA. Previous works have shown that FSA differ at different speeds [42] and that FSA changes during acceleration (compared to steady state running [43]. 

### 4.3. Foot Strike Pattern

Previous observations of FSP during a race have been made using high-speed video cameras, providing only a snapshot of one, or a few, points in time, and may be missing information that occurs throughout the run [10,13,21,22,28]. The present study directly evaluated FSP during overground running with the use of a functional IMU, providing a measure of FSP throughout the entire 800 m run. Previous observations of FSP over varying distances have shown changes from a NRF to RF strike pattern throughout a race or hard run leading us to believe there would be a change in FSP over the course of the 800 m [8,21,22,26,44]. This study found FSP, measured as the percentage of foot strikes being RF, did not change significantly over the course of the 800 m run on an outdoor track.

Former studies looking at changes in FSP throughout a run using videography have been done in longer distances than an 800 m run. For example, in a marathon, it has been observed that the percentage of NRF strike runners declines as the race progresses, with many of those runners switching to a more posterior landing [10,21,22]. Jewell and colleagues observed FSP during a 15–20-min fatiguing run on a treadmill and found a transition from FF to a more MF strike pattern [26]. In track race scenarios, inferences about changes in FSP can be made from other evaluated variables. Elliot and Ackland used high-speed video cameras during the 10,000-m race and found runners tended to decrease the relative backwards velocity of the ankle at foot strike, creating a greater likelihood of a RF strike in these elite runners [44]. In a study of elite 800 m and 1500 m runners, an increase in ground contact time throughout the race was observed [8]. Although changes in FSP over the course of the race were not monitored, the increase in ground contact time could be indicative of a shift to striking more posteriorly on the foot [8]. These studies did not directly measure FSP, so it is unknown if changes in FSP occurred during these previous investigations of shorter distance races. 

Although there has been evidence to suggest shifts in FSP in some intense running or races, our results did not support this finding. Other previous investigations have also shown no changes in FSP throughout a hard effort or race. No kinematic changes have been observed in repeated high intensity 100 m or 400 m runs [27,45]. Similarly, in a marathon race, it was shown the majority of runners did not change their FSP throughout the race [28]. These conflicting results leave questions unanswered for the reasons why some runners may change their FSP throughout a race and others do not. In our sample, none of the runners gradually altered their FSP from NRF to RF strike throughout the 800 m. Some variation did occur throughout the race for most runners; however, these changes were not large enough to elicit an overall change in FSP, and most of these variations were in relation to whether the participant was running on a curve or straight portion of the track. 

### 4.4. Limitations

Our observations were limited to analyzing the right foot during this 800 m run, however, there is evidence to suggest that the left foot is also affected by running on curves. Where the right foot has been shown to exhibit greater supination on the curves than on the straights, the left foot has been shown to be in a pronated position at touchdown [38]. The focus of this analysis was on sagittal plane motion of the foot via the attached sensor. Future work could explore gyroscope data in all three planes, adding inversion/eversion and adduction/abduction motion, which would allow for a more complete understanding of foot movement throughout a race. This study was performed outside, and the weather may have affected the participants’ running ability; however, weather is not controllable on an outdoor track in a racing environment. It was impossible to simulate a true race environment with other competitors and fans that lead to better performances; however, verbal encouragement was provided in an attempt to overcome this barrier but is a limitation of our approach. We had participants run in their regular training shoes rather than a racing flat or spike, possibly affecting our foot strike data, but most likely would have provided an even more pronounced NRF strike pattern. 

## 5. Conclusions

The purpose of this study was to use an IMU on the foot to evaluate FSP during a maximal 800 m run. Contrary to our hypothesis, FSP did not appear to change consistently over the course of this 800 m race, with participants remaining in their selected FSP throughout the run. However, participants did show fluctuations in FSA on the curved portions of the track compared to the straights. Running on the curves of the track produced significantly decreased FSA compared to the straights, supporting previous investigations into curved running [39]. The use of FSA may allow for the detection of subtle changes in foot strike characteristics, which is not possible with FSP classifiers such as RF and NRF. This study was the first to use an IMU to measure FSP continuously throughout an 800 m run, allowing for quantification of running characteristics unobtrusively in a real-world scenario. 

## Figures and Tables

**Table 1:** Participant characteristics (mean ± standard deviation).

	Females (14)	Males (7)
Age (years)	24.43 ± 3.92	24.71 ± 5.06
Height (cm)	166.83 ± 7.29	181.53 ± 7.48
Mass (kg)	58.49 ± 7.06	72.27 ± 8.53
Miles per Week	33.75 ± 18.10	42.14 ± 22.89

## Data Availability

The data presented in this study will be made available upon request—please contact corresponding author.

## Supplementary Materials

The following are available online at https://www.mdpi.com/article/10.3390/s21175782/s1, Table S1: Subject characteristics, race time and foot strike pattern (% RF strike) throughout the race—ordered by finishing time (fastest to slowest), Document S1: Data Processing Steps
