# Supplementary material for: Continuous Tracking of Foot Strike Pattern during a Maximal 800-Meter Run
Source: Sensors (Basel). 2021 Aug 27;21(17):5782. doi: 10.3390/s21175782 (PMC8434103; doi:10.3390/s21175782)
Supplement: Supplementary file 1 [file sensors-21-05782-s001.zip › Document S1 - Data Processing Steps.pdf]

S2 - Data Processing Steps

1. Import gyroscope and accelerometer data (from sensor) to Matlab
2. Import 100 m interval times from stopwatch data (via Excel)
3. Integrate sensor gyroscope data (sagittal plane) to find continuous sensor (foot) angle
4. Plot image of sensor (foot) angle data and using visual inspection find time when subject starts to run (before start: angle data constant, at start abrupt change in angle value)
5. Align this time (IMU start time) with stopwatch start time
6. Create 100 m interval measures for accelerometer and gyroscope data (from alignment with stopwatch interval times)
7. Find stride intervals:
   - Using sensor (foot) angle data, the maximum angle which occurs periodically (at each stride) aligns with the time when the foot is in the air, roughly at middle of aerial phase. The interval in-between these maximum angles is where foot strike occurs and where FSP (Foot Strike Pattern) and FSA (Foot Strike Angle) analyses are done
8. Do analysis of FSP and FSA in all intervals between maximum sensor (foot) angles:
   - FSP:
     i. Find timepoint of maximum resultant acceleration: t_{Amax}
     ii. Find timepoint 15 ms later: t_{+15ms}
         1. Calculate average angular velocity, ω_{avg}, between t_{Amax} and t_{+15ms} (from sagittal plane gyroscope data)
         2. From this value determine FSP
            a. $\omega_{avg} > 0$: RF
            b. $\omega_{avg} < 0$: NRF
     iii. Repeat for all foot strikes in each 100 m interval
     iv. Calculate $FSP_{\%RF}$ as number of FSP that were rearfoot compared to all foot strikes in each 100 m interval
   - FSA:
     i. Find timepoint of maximum resultant acceleration: t_{Amax}
     ii. Find timepoint when foot is stationary on the ground (50 ms interval after t_{Amax} when foot has lowest mean resultant acceleration): t_{stat}
     iii. Calculate FSA: $\theta_{tAmax} – \theta_{tstat}$ (from sagittal plane (foot) angle data)
     iv. Repeat for all foot strikes in each 100 m interval
     v. Average FSA over 100 m intervals

* For more details please see: van Werkhoven, H.; Farina, K.A.; Langley, M.H. Using A Soft Conformable Foot Sensor to Measure Changes in Foot Strike Angle During Running. *Sports* **2019**, *7*, 184, doi:10.3390/sports7080184.
