# Supplementary material for: Continuous Tracking of Foot Strike Pattern during a Maximal 800-Meter Run
Source: Sensors (Basel). 2021 Aug 27;21(17):5782. doi: 10.3390/s21175782 (PMC8434103; doi:10.3390/s21175782)
Supplement: Supplementary file 1 [file Table S1 - Subject Characteristics, Race Time and Foot Strike Pattern.pdf › Table S1 - Subject Characteristics, Race Time and Foot Strike Pattern.pdf]

Table S1 – Subject characteristics, race time and foot strike pattern (% RF strike) throughout the race – ordered by finishing time (fastest to slowest).

Subject Number	Height (m)	Mass (kg)	Sex (M/F)	Final Time (s)	$FSP_{\%RF}$ 100 m	$FSP_{\%RF}$ 200 m	$FSP_{\%RF}$ 300 m	$FSP_{\%RF}$ 400 m	$FSP_{\%RF}$ 500 m	$FSP_{\%RF}$ 600 m	$FSP_{\%RF}$ 700 m	$FSP_{\%RF}$ 800 m	$FSP_{\%RF}$ Race Ave
**S1**	169.9	63.6	M	128.35	8%	4%	0%	4%	0%	0%	0%	0%	2%
**S2**	184.5	63.6	M	128.75	0%	4%	4%	40%	8%	28%	8%	46%	17%
**S3**	188.7	77.8	M	139.92	0%	0%	0%	0%	0%	0%	0%	0%	0%
**S4**	184.0	71.6	M	144.10	0%	0%	0%	0%	0%	0%	0%	0%	0%
**S5**	172.0	65.9	M	146.25	4%	0%	0%	0%	0%	0%	0%	0%	0%
**S6**	187.6	77.4	M	146.75	0%	0%	0%	0%	0%	0%	0%	0%	0%
**S7**	164.9	61.6	F	153.91	0%	0%	0%	0%	0%	0%	0%	3%	0%
**S8**	165.0	54.0	F	156.11	0%	0%	0%	0%	0%	0%	0%	4%	0%
**S9**	163.5	53.9	F	156.13	3%	0%	0%	9%	0%	28%	0%	19%	7%
**S10**	168.6	48.2	F	156.36	0%	0%	0%	0%	0%	0%	0%	0%	0%
**S11**	181.5	65.8	F	157.26	0%	0%	0%	0%	0%	0%	0%	0%	0%
**S12**	162.5	55.6	F	160.70	7%	8%	0%	0%	0%	0%	0%	6%	3%
**S13**	157.0	50.3	F	161.92	97%	100%	100%	100%	100%	100%	100%	100%	100%
**S14**	177.0	63.1	F	163.47	39%	69%	24%	37%	3%	22%	0%	17%	26%
**S15**	161.0	50.8	F	165.80	45%	84%	100%	100%	100%	100%	100%	100%	91%
**S16**	167.9	66.7	F	173.28	91%	96%	51%	91%	14%	81%	24%	77%	66%
**S17**	163.6	56.7	F	182.46	97%	100%	100%	100%	100%	100%	100%	100%	100%
**S18**	161.6	57.7	F	194.11	97%	100%	100%	100%	100%	100%	100%	100%	100%
**S19**	184.0	86.0	M	203.20	0%	0%	0%	0%	0%	0%	0%	0%	0%
**S20**	178.9	72.6	F	203.99	100%	100%	100%	100%	100%	100%	100%	100%	100%
**S21**	162.6	61.9	F	216.04	10%	0%	0%	0%	0%	0%	0%	0%	1%
